# Enhanced recovery of low concentration protein and peptide solutions on ultra-low binding microplates

**DOI:** 10.4155/fsoa-2018-0099

**Published:** 2019-01-25

**Authors:** Christopher M Weikart, Adam P Breeland, Ahmad H Taha, Brian R Maurer

**Affiliations:** 1 SiO_2_ Medical Products, Inc., 350 Enterprise Drive Auburn, AL 36832 USA

**Keywords:** microplates, nonspecific protein binding, protein and peptide adsorption

## Abstract

SiO_2_ Medical Products (SIO) developed PureWARE™ Ultra-Low Binding (ULB) plasma-treated microplates with the combined benefits of enhanced protein recovery and reduced extractables. This study demonstrates enhanced protein recoveries, but at ten-times lower protein concentration, or 0.1 nM, compared with a prior study. In addition, no significant effect on enhanced protein recovery of plasma-treated microplates was observed in a long-term stability study carried out for 26 months under ambient storage conditions. Furthermore, recovery of three different peptide solutions, in the concentration range of 1.5–12 nM, was also shown to be enhanced on plasma-treated microplates relative to standard polypropylene microplates.

## Lay abstract

Testing of therapeutic biologic drugs, called bioanalysis assays, is targeted at understanding how the drug is metabolized by the body and its safety. Assays are conducted in a polymer microplate that has many mini-wells, like miniature test tubes, that contain drug solution for testing. Biologic drug molecules are notorious for sticking to the polymer surface. If the amount of biologic in the liquid of a microplate well is too low, there will be nothing left in solution to test. A new coated microplate was developed to enable assays using very low concentrations of biologic molecules with little to no sticking.

The development of next-generation therapeutic protein and peptide drugs depends upon rigorous bioanalysis assays that are employed throughout discovery, preclinical and clinical trials. Microplates are used extensively for high throughput bioanalysis assays involving preparation, storage and detection of therapeutic drug molecules in aqueous solution. While there are many challenges in bioanalytical testing, arguably one of the most unpredictable, time-consuming and ubiquitous is nonspecific binding or the adsorption of the therapeutic drug to the walls of the microplate well.

Most standard microplates on the market today are composed of polymers such as polypropylene and polystyrene, which tend to exhibit moderately hydrophobic or water repelling surface characteristics. A particularly dominant adsorption mechanism is the ‘hydrophobic effect’, whereby strong interactions are established between hydrophobic amino acid constituents of protein and peptide molecules and the hydrophobic surface of standard polymeric microplates. Protein and peptide chemical characteristics, including polarity, structure, folding kinetics, charge, and size, can give rise to other mechanistic adsorption pathways [1] that can enhance their stickiness to polymeric surfaces. Irrespective of the adsorption mechanisms, loss of the target drug molecules in solution is exacerbated as the concentration decreases. Below a critical concentration, most or all the protein or peptide can be lost to adsorption with nothing left in solution to detect or analyze. Reports [2–4] of peptide loss due to adsorption on plastic and glass laboratory consumables is arguably just as prevalent as proteins. Workarounds to circumvent peptide and proteins losses, including protein blocking agents, surfactants and siliconizing agents, are plentiful, but no single approach is universal, with unpredictable results and undesirable trade-offs.

SiO_2_ Medical Products (SIO) first reported [5] on the development of new PureWARE™ ultra-low binding (ULB) plasma-treated microplates with the combined benefits of high protein recovery and low extractables. A proprietary microplate plasma treatment technology enabled high protein recoveries down to 1 nM in concentration and compared favorably to existing low-protein-binding microplates on the market. The current study explores the recovery of proteins in solution down to 0.1 nM concentration and 2 years of shelf-life testing on plasma-treated microplates. Additionally, the recovery of three common peptides was also explored and compared with standard polypropylene and commercial low-bind microplates on the market.

## Materials & methods

### Materials

Five AlexaFluor 488 dye-labeled protein conjugates, all purchased from ThermoFisher (Molecular Probes, OR, USA), were selected based on their broad range of molecular weights, isoelectric points and other characteristics. The proteins included bovine serum albumin (BSA), human fibrinogen (FBG), bacterial protein A (PrA) and protein G (PrG), and human transferrin (TFN). An aqueous, phosphate-buffered saline solution of pH 7.4 (Sigma-Aldrich, MO, USA) was used as a medium for all protein solutions except for FBG, which was dissolved in a sodium bicarbonate buffer solution at pH 8.3 (Sigma-Aldrich).

Peptide analysis included AlexaFluor 488 dye-labeled insulin conjugate purchased from Nanocs via Fisher Scientific, AlexaFluor 488 dye-labeled glucagon conjugate purchased from Anaspec Inc., Fremont, CA, USA and AlexaFluor 488 dye-labeled EGF conjugate purchased from ThermoFisher (Molecular Probes).

### Microplates

Microplates were injection-molded from polypropylene homopolymer resin in a class 7 cleanroom at SIO's manufacturing plant in Auburn, AL, USA. PureWARE ULB plasma-treated microplates are in some cases referred to as simply ‘plasma-treated’, and SIO microplates without plasma treatment are referred to as ‘standard polypropylene’ hereon. Benchmark low-protein-binding polypropylene microplates are referred to herein as Eppendorf Protein LoBind^®^ or Eppendorf LoBind microplates. The format of all microplates used in this study are deep 96-well (500-μl well volume) unless specified otherwise.

### Fluorescence spectroscopy

Protein and peptide recovery in microplates were determined using fluorescence spectroscopy with a BioTek Synergy H1 microplate reader [6]. The fluorescence intensity of a known concentration of AlexaFluor 488-labeled protein (pH 8.3 for FBG, pH 7.4 for all other proteins and peptides) was measured and then added to a series of wells and monitored over various incubation times; specifically, filled microplates were stored in the absence of light at room temperature for 4, 24, 72 and 96 h. All recovery results are reported after 24 h of incubation because protein and peptide adsorption occur quickly with the first 4 h and most certainly before 24 h. This is an indication that the surface binding sites of the PureWARE ULB microplates become saturated quickly and no further adsorption loss after 4 h. At each incubation time point, protein and peptide solutions from four wells were transferred from the test microplate to a black 96-well flat-bottom read plate and the fluorescence intensity of each well was again measured via the microplate reader. The fluorescence measurement signals from protein and peptide solutions are blank corrected by filling wells with phosphate buffer solution and subtracting the average signal from the initial and final concentrations of each well.

Protein and peptide recovery, expressed as percent recovery, was calculated from the ratio of the fluorescence intensity of the test solution after sample incubation to the initial solution's intensity. This experiment was conducted by pipetting working solution from the preparation vessel directly to the read plate for the initial intensity and then immediately filling the test microplate with the same working solution. After a prescribed residence time in the sample microplate, the test solution was transferred to the read plate and the fluorescence intensity measured and recorded. It was confirmed during method development that protein and peptide loss from adsorption to pipette tips was negligible.

### Plasma treatment process

The advanced plasma treatment process employed by SIO comprised a vacuum system, a gas delivery system and a power delivery system. The vacuum system was composed of a vacuum chamber and pump, whereby the process pressure was in the range of 1–3 Torr. The gas delivery system was composed of mass-flow controllers to deliver oxygen gas and water vapor. The power delivery system was modulated at a radiofrequency (13.56 MHz). A capacitively coupled electrode system was composed of an outer cylindrical electrode and an inner counter-electrode separated by a ceramic dielectric. The counter-electrode doubled as a gas delivery tube into the vacuum chamber. Ten 96-well 500-μl microplates were plasma-treated together in one batch. No additional treatment or washing of the plasma-treatment microplates was performed.

## Results & discussion

### Protein recovery at low concentrations

A technical study published by Weikart *et al*. [5] examined the dependence of protein recovery on protein concentration in aqueous solution using PureWARE ULB 96-well 500-μl plasma-treated microplates and Eppendorf LoBind microplates. Mean recoveries for three different protein solutions incubated in SIO plasma-treated microplates were at least 90% and practically independent of concentration in the range of 1–12.5 nM. FBG, a particularly sticky protein, exhibited a mean recovery approaching 85% in a 1 nM solution incubated on a SIO plasma-treated microplate compared with 10% on a standard polypropylene microplate. The improved recovery of all proteins was attributed to the tailored surface characteristics of the polypropylene microplates by a plasma treatment process. The treatment itself is an oxidizing plasma of oxygen and water vapor generated in a vacuum chamber filled with a batch of microplates. The resulting microplate conforms to four general surface characteristics [5] that resist protein adsorption; hydrophilic, presence of hydrogen bond acceptor groups, absence of hydrogen bond donor groups, and charge neutral. These general guidelines were first proposed by Whitesides *et al*. [7–9] that resulted from comprehensive protein adsorption studies on a broad range of surface chemistries.

The protein bioanalysis group at Merck conducted a separate independent study that compared the recovery of isotope-labeled monoclonal antibody formulations on PureWARE ULB microplates and Siliguard™ low-bind microplates [10]. The results of this study showed that both microplates exhibited similar monoclonal antibody recovery at all concentrations with the exception of the lowest concentration (0.1 μg/ml) whereby the PureWARE ULB microplates had higher recovery. Furthermore, the PureWARE ULB microplates exhibited more consistent recovery at every concentration.

**Figure 1.**
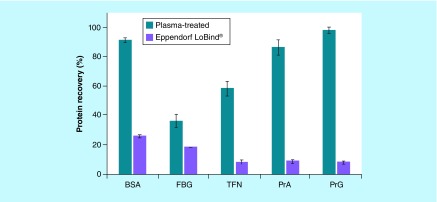
**A comparison of protein recovery between two low bind microplates using 0.1 nM solutions of five model proteins.** Recovery of five model dye-labeled proteins from their 0.1 nM solutions at pH 7.4 (except for pH 8.3 for FBG) after a 24-h incubation at room temperature under ambient air in PureWARE™ ultra-low binding plasma-treated polypropylene microplates and in Eppendorf Protein LoBind^®^ microplates examined for comparison (all were deep 96-well, 500-μl microplates). All experiments were carried out in triplicate and the bars correspond to the mean values with standard deviations shown. BSA: Bovine serum albumin; FBG: Fibrinogen; PrA: Protein A; PrG: Protein G; TFN: Transferrin.

Experiments in this study were designed to check if protein recovery remains independent of protein concentration in solution down to 0.1 nM or ten-times lower than the prior study [5]. Results in Figure 1 shows that three proteins, (i.e., BSA, PrA and PrG), exhibit mean protein recoveries at least 90% for the PureWARE ULB plasma-treated microplates. Eppendorf LoBind microplates exhibited significantly lower protein recovery (i.e., less than 30%) at 0.1 nM concentration. This result is attributable to more protein lost from nonspecific protein binding or adsorption on the walls of the Eppendorf LoBind microplates as compared to the SIO plasma-treated microplates. Recoveries for the stickier FBG and TFN proteins at 0.1 nM (Figure 1) were significantly higher and approaching 40% and 60%, respectively, for SIO plasma-treated microplates compared to Eppendorf LoBind.

### Storage stability of plasma-treated microplates

**Figure 2.**
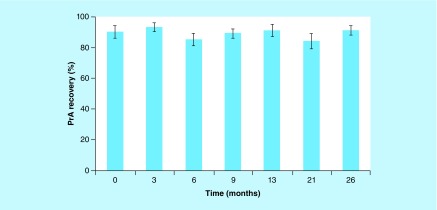
**Shelf-life stability of protein A recovery on PureWARE™ microplates stored for over 2 years at ambient temperature.** Shelf life stability of PrA recovery (2 nM) on PureWARE™ ultra-low binding plasma-treated 96-well microplates (500 μl) after 26 months of storage at ambient temperature (i.e. 25°C) in the dark. The mean recovery and error bars represent the average of all 96 wells from two separate microplates. PrA: Protein A.

Protein recovery of five different 12 nM protein solutions incubated on PureWARE ULB 96-well 500-μl plasma-treated microplates was shown to be stable up to 9 months of simulated shelf storage at ambient temperature conditions (i.e., 25°C) in a prior study [5]. Since this result was first reported, protein PrA recovery stability has been continually monitored for over 2 years on 96-well 500-μl plasma-treated polypropylene microplates stored under similar conditions. Protein recovery results in the range of 84–93%, shown in Figure 2, are maintained for up to 26 months of ambient storage on plasma-treated microplates. Although these protein recovery results are based on microplates stored in the dark, results not reported here are equally stable stored under laboratory lighting conditions. Furthermore, 96-well 1000-μl and 384-well 120-μl plasma-treated microplates exhibited comparable enhanced protein recovery stability (not reported here) under similar ambient storage conditions.

### Peptide recovery

Both peptides and proteins are constructed from chemically bonded strings of amino acids. Peptides are distinguished from proteins based on their molecular size, which is derived from the number of amino acids in their molecular architecture. For example, peptides are generally composed of 50 or fewer amino acids and molecular weights of about 6 kDa or less. Proteins, by comparison, are composed of more than 50 amino acids, and thus, much larger molecules. Larger proteins, such as FBG (340 kDa), tend to be stickier or higher adsorbing compared with smaller proteins because more sites of contact can be established with the surface. Exceptions, such as hemoglobin (65 kDa), exhibit more adsorption compared with FBG because of the sequence and chemistry of its amino acid constituents. Peptides are no different in their complex and sometimes unpredictable adsorption behavior.

**Figure 3:**
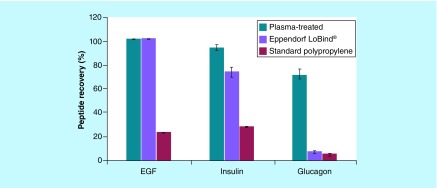
**A comparison of peptide recovery on two low bind microplates using 1.5 nM solutions of three model peptides.** Recovery of three-model dye-labeled peptides from their 1.5 nM solutions at pH 7.4 (except for pH 8.3 for FBG) after a 24-h incubation at room temperature under ambient air in standard polypropylene microplates, PureWARE™ ultra-low binding plasma-treated polypropylene microplates and in Eppendorf LoBind^®^ microplates examined for comparison (all were deep 96-well, 500-μl microplates). All experiments were carried out in triplicate, and the bars correspond to the mean values with standard deviations shown. FBG: Fibrinogen.

The three peptides used in this study have relatively similar molecular weights with the largest being EGF at 6.2 kDa and the smallest being glucagon at 3.5 kDa. Despite this similarity, the recovery of these peptides in 1.5 nM solutions, albeit low, varies significantly on a standard polypropylene microplate as shown in Figure 3. Similar to proteins, peptide's unique sequence and characteristics of amino acid constituents impacts the overall adsorption to surfaces.

Both PureWARE ULB plasma-treated and Eppendorf LoBind microplates exhibited 100% EGF peptide recovery in a 1.5 nM solution compared with the standard polypropylene microplate recovery of 20% shown in Figure 3. Insulin recovery was also improved with both plasma-treated and Eppendorf LoBind microplates at 94 and 74%, respectively. Only plasma-treated microplates exhibited an improved glucagon recovery of 71% compared with the standard polypropylene microplates. The Eppendorf LoBind microplates showed practically no improvement in glucagon peptide recovery compared with the standard microplate.

**Figure 4.**
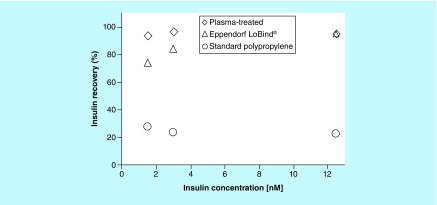
**A comparison of insulin recovery as a function of concentration on low bind microplates.** Recovery of insulin as a function of concentration after 24-h incubation at room temperature in PureWARE^TM^ ultra-low binding plasma-treated 96-well (500 μl) microplates, standard polypropylene microplates and Eppendorf LoBind^®^ microplates. The mean recovery and error bars represent the average of eight replicate wells on two separate microplates at each concentration.

**Figure 5.**
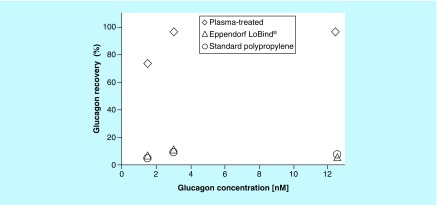
**A comparison of glucagon recovery as a function of concentration on low bind microplates.** Recovery of glucagon as a function of concentration after 24-h incubation at room temperature in PureWARE™ ultra-low binding plasma-treated 96-well (500 μl) microplates, standard polypropylene microplates and Eppendorf LoBind^®^ microplates. The error bars represent the average of eight replicate wells on two separate microplates at each concentration.

The dependence of insulin and glucagon recovery on peptide concentration over the range of 1.5 to 12.5 nM and incubated in 96-well 500-μl microplates is shown in Figures 4 and 5, respectively. Insulin recovery was maintained at approximately 95% and remained practically independent of concentration on the plasma-treated microplates (Figure 4). Eppendorf LoBind microplates exhibited a 95% insulin recovery at 12.5 nM concentration but drops to 74% as the concentration approached 1.5 nM.

Glucagon recovery on plasma-treated microplates was maintained above 95% down to 3 nM concentration then dropped to 70% approaching 1.5 nM, as shown in Figure 5. Eppendorf LoBind microplates showed glucagon recovery that was essentially the same as standard microplates in the range of 5–10% over the entire concentration range of 1.5–12 nM. The PureWARE ULB plasma-treated microplates exhibited a significant peptide recovery advantage for insulin, and especially glucagon, solutions compared with competitive microplates.

## Conclusion

In summary, PureWARE ULB plasma-treated polypropylene microplates were used to demonstrate the enhanced recovery of five model proteins down to 0.1 nM concentration compared with a commercial low-protein-bind microplate on the market. Enhanced protein recovery was shown to be stable over 2 years of storage at ambient temperature and insensitive to lighting conditions. Enhanced recovery of three model peptides was exhibited on similar SIO plasma-treated microplates over 1.5–12 nM concentration range. Insulin, and especially glucagon, recovery was particularly advantaged compared with a commercial low-bind microplate.

## Future perspective

The introduction of high-throughput screening systems in the 1990s and the increase of well densities, which miniaturized well volumes on microplates has significantly reduced the cost per assay. Further improvements in analytical instrument performance have resulted in better sensitivity, greater detection range, lower limits of detection and reduced assay time. While there have been significant advances in microfluidic devices such as ‘lab-on-a-chip’, and surface plasmon resonance immunoassays, these technologies have not displaced high-throughput screening system microplate assays to date. The integration of smart phone technology with novel paper immunoassay technology could indeed be the future to further reduce cost and extend the outreach to developing countries.

**Summary points**

- PureWARE™ Ultra-Low Binding microplates exhibited enhanced recovery of five model proteins down to 0.1 nM concentration compared with a commercial low-protein-bind microplate on the market.
- Enhanced protein recovery was shown to be stable over 2 years of storage.
- Enhanced recovery of three model peptides was exhibited over 1.5–12 nM concentration range.
- Both insulin and especially glucagon recovery was particularly advantaged compared with a commercial low-bind microplate.
- PureWARE Ultra-Low Binding microplates exhibit low adsorption and high recovery characteristics for some of the stickiest proteins (e.g., fibrinogen) and peptides (e.g., glucogon).
- This technology enables the use of low concentrations of high value therapeutic drugs for evaluating their biological efficacy.
